# Patterns and drivers of macro- and micro-diversity of mudflat intertidal archaeomes along the Chinese coasts

**DOI:** 10.1128/msystems.01434-25

**Published:** 2026-03-27

**Authors:** Yan Li, Mengzhi Ji, Qichao Tu

**Affiliations:** 1 Institute of Marine Science and Technology, Shandong University 520252, Qingdao, China; 2 Shandong Key Laboratory of Intelligent Marine Engineering Geology, Environment and Equipment, Qingdao, China; 3 Southern Marine Science and Engineering Guangdong Laboratory (Zhuhai), Zhuhai, China; Stellenbosch University, Stellenbosch, South Africa

**Keywords:** intertidal archaeomes, macro-diversity, micro-diversity, horizontal gene transfer, spatial scaling, community assembly

## Abstract

Archaea are widespread in Earth’s ecosystems, contributing to ecosystem multifunctioning and stability. Compared to bacteria, our understanding of the biodiversity and underlying drivers of archaeal communities in representative ecosystems remains much less tapped. In this study, the macro- and micro-diversity of mudflat intertidal archaeomes were comprehensively analyzed at a large geographic scale, aiming to resolve the ecological drivers determining the variations in archaeal biodiversity. The compositions of mudflat intertidal archaeal taxa highly varied, especially the dominant *Thaumarcheota* and *Euryarchaeota*, but maintained relatively stable functional potential across space, demonstrating that functional traits were selected by the ecosystem in priority. While archaeal communities carried important functional traits mediating various biogeochemical cycling processes, horizontal gene transfer played critical roles in endowing functional genes for many archaeal lineages, such as the citric acid cycle in *Methanosarcinia* and various amino acid metabolism genes in *Thermoplasmata*. Spatial scaling, including latitudinal diversity gradient and distance-decay patterns (DDR), was clearly observed for archaeal taxonomic groups, but only DDR was weakly observed for functional traits. Intra-population genetic variations were significantly and positively associated with community macro-diversity, demonstrating covariations between nucleotide-level micro- and community-level macro-diversity. The compositions of intertidal archaeomes were mainly structured by homogeneous selection, with different phylogenetic bins being shaped by distinct ecological processes and remarkable variations across different sites. The study contributes to a comprehensive insight into the mechanisms shaping archaeal diversity and ecological characteristics within a fluctuating ecosystem.

**IMPORTANCE:**

The dynamic intertidal mudflat ecosystems host intense biogeochemical activities mediated by microbial communities, among which archaea contribute as an essential component but remain much less understood compared to bacteria. To gain better insights into the diversity, functional potential, and ecological drivers of archaeal communities in intertidal mudflats, archaeal phylogenetic signatures and genomic sequences were recovered via amplicon sequencing of 16S rRNA genes and shotgun metagenomes, targeting both macro- and micro-diversity. The results showed that archaeal taxonomic composition highly varied across space, whereas the functional potential remained relatively stable. Horizontal gene transfer served as an important source of archaeal metabolic diversity, obtaining additional genes linked to key biochemical pathways. The dominance of environmental selection further demonstrated the ecological forces governing archaeal communities in highly variable coastal habitats. This study established a large-scale framework for understanding the microbial ecology of intertidal archaeomes in dynamic coastal ecosystems.

## INTRODUCTION

Archaea are widely distributed in the Earth’s biosphere, including natural, host, and artificial environments (1, 2). As a critical component of the Earth’s microbiome, they mediate various biogeochemical processes of essential elements, particularly the cycling of carbon (C), nitrogen (N), and sulfur (S) (3). For instance, ammonia-oxidizing archaea (AOA) are major contributors to nitrification, which oxidizes ammonia to nitrite (4, 5). As an essential connector of the carbon and nitrogen cycles, denitrifying anaerobic methane oxidation (DAMO) plays a major role in anoxic ecosystems. It is estimated that DAMO contributes to 65% to 100% in removing the total anaerobic methane in intertidal zones, making it an important methane sink (6). Methanogenic archaea produce methane under anaerobic conditions from substrates such as CO_2_, acetate, and other fermentation products. Notably, archaeal methanogenesis accounts for more than half of Earth’s biogenic methane (7, 8). In addition, dissimilatory sulfate reduction (DSR), predominantly driven by bacterial lineages, has also been observed in certain members of *Crenarchaeota* independent of Archaeoglobus (9).

Although progress has been continuously made, there remains a considerable gap in our comprehension of the biological characteristics, ecological significance, and functional potential of archaeal assemblages in various ecosystems, mainly due to the difficulties in isolating and culturing archaeal species. Approximately 66% of the archaeal lineages in the 26 known phyla lacked representative cultivated species (10). Over the past few years, remarkable insights have been gained by recovering archaeal genomic sequences from shotgun metagenomes, especially via metagenome-assembled genomes (MAGs). Archaeal MAGs have been recovered in many representative ecosystems such as terrestrial geothermal springs (11), hydrothermally heated sediment (12), the polar Arctic Ocean (13), and the human gut (1), demonstrating unique ecological patterns and functions in these ecosystems. Comparative analyses of complete genomes and MAGs of Asgard archaea have revealed novel phyla such as *Wukongarchaeota* and, at the same time, demonstrated the potential origin of eukaryotes from within Asgard branches (14). The information gleaned from these excavations not only supplements the metabolic pathways and characteristics of uncultivable archaea but also provides insights into the complex evolutionary relationship of Earth life forms.

As one of the most essential components in ecology, the biodiversity of biological communities encompasses both macro- and micro-scales (15) and multiple dimensions including taxonomic, genetic, phylogenetic, and functional (16). Among these, macro-diversity refers to the community diversity across different scales, such as local (α-diversity), between-site (β-diversity), and regional (γ-diversity), whereas micro-diversity refers to nucleotide-level intra-population variations, such as single-nucleotide polymorphisms (SNP), nucleotide diversity, and selective pressure (17, 18). Consequently, by crosslinking different scales and dimensions, the scheme of biodiversity becomes multifaceted. Additionally, the dimensions of biodiversity (e.g., taxonomic, genetic, phylogenetic, and functional) are closely associated with those of micro-diversity, making the issues of biodiversity more complex. Effort has been made to disentangle the ecological patterns of archaeal macro-diversity across various ecosystems (19, 20). By employing sophisticated community assembly analysis methods, the mechanisms controlling archaeal communities in various ecosystems and their ecological adaptations amid environmental fluctuations are addressed (21). Within a grassland soil ecosystem, the importance of stochastic processes shaping archaeal communities gradually wanes over time as experimental warming progresses (21). Considering their close relationship in the biodiversity scheme, it is therefore of necessity to investigate both the macro- and micro-diversity for microbial communities when approachable.

The intertidal zone is the transitional area located between land and sea, periodically exposed to air at low tide and submerged under seawater at high tide. It forms one of the largest and most dynamic coastal habitats (22). The intertidal mudflats are highly variable environments shaped by tides, waves, storms, and recurrent cycles of erosion and accretion (23). Such strong and frequent physical disturbances create pronounced environmental gradients and rapidly changing sedimental conditions, which in turn support exceptionally active microbial processes of both prokaryotes and micro-eukaryotes (23). The intertidal microbial communities, in which archaea comprise a critical component, are uniquely adapted to the fluctuations, playing crucial ecological roles including nutrient cycling and the purification of multiple pollutants (24). Here, archaeal communities residing in the mudflat intertidal zones along the coastal regions of China were investigated using culture-independent methods. The following ecological questions focusing on both macro- and micro-diversity were addressed: (i) How diverse are intertidal archaeomes at both macro- and micro-scales and at different dimensions? (ii) How are intertidal archaeomes composited along the Chinese coasts? (iii) How are different archaeal assemblages and lineages structured by stochastic and deterministic processes? The results demonstrated high archaeal biodiversity in the intertidal zones, carrying important functional genes involved in various biogeochemical cycling. Frequent horizontal gene transfer (HGT) events were observed for the recovered archaeal genomes, mainly occurring within the same phyla. The archaeal taxonomic composition highly varied spatially but maintained stable functional potential. Macro- and micro-diversity were strongly associated, with close interconnected relationships between these two. This study offered valuable insights into the macro- and micro-diversity of intertidal archaeomes.

## RESULTS

### Overall archaeal diversity in intertidal mudflats

**Fig 1:**
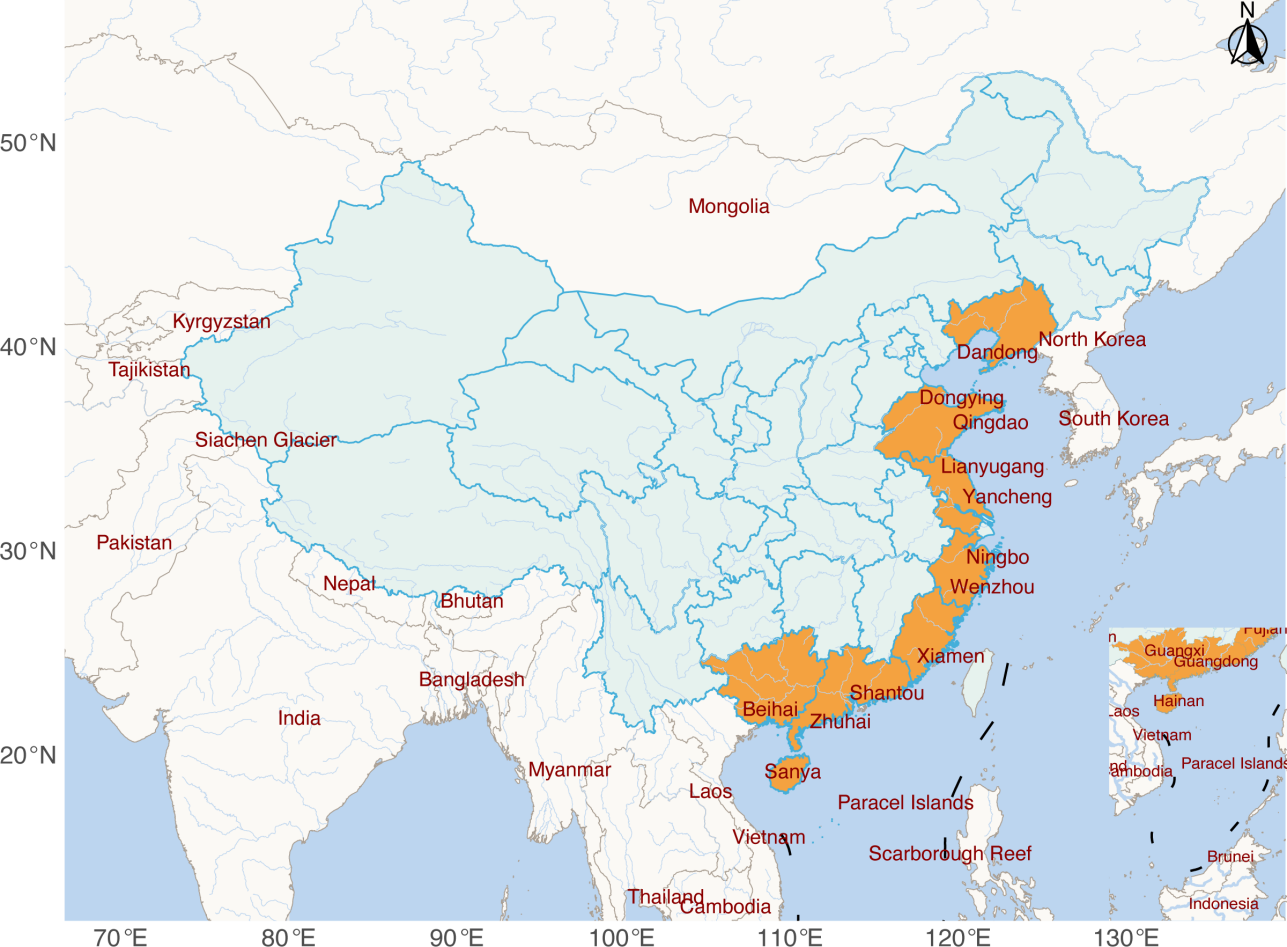
Geographic locations of intertidal samples collected in the intertidal zones of China. A total of 144 samples were collected from 12 intertidal mudflat habitats along the coastal areas of China spanning from SY to DD (Data S1).

**Fig 2:**
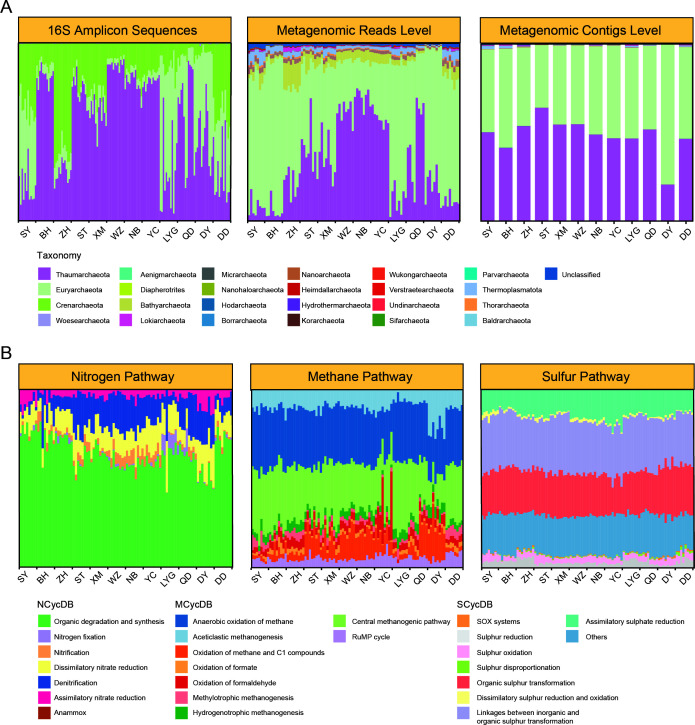
Taxonomic and functional composition of intertidal archaeomes along the Chinese coasts. (**A**) Taxonomic composition of intertidal archaeal communities at the phylum level revealed by amplicon sequencing, shotgun metagenomic reads, and assembled shotgun metagenomic contigs across different sampling locations. (**B**) Microbial functional genes involved in nitrogen, methane, and sulfur cycling based on NCycDB, MCycDB, and SCycDB, respectively. Functional compositions are shown at the pathway level.

**Fig 3:**
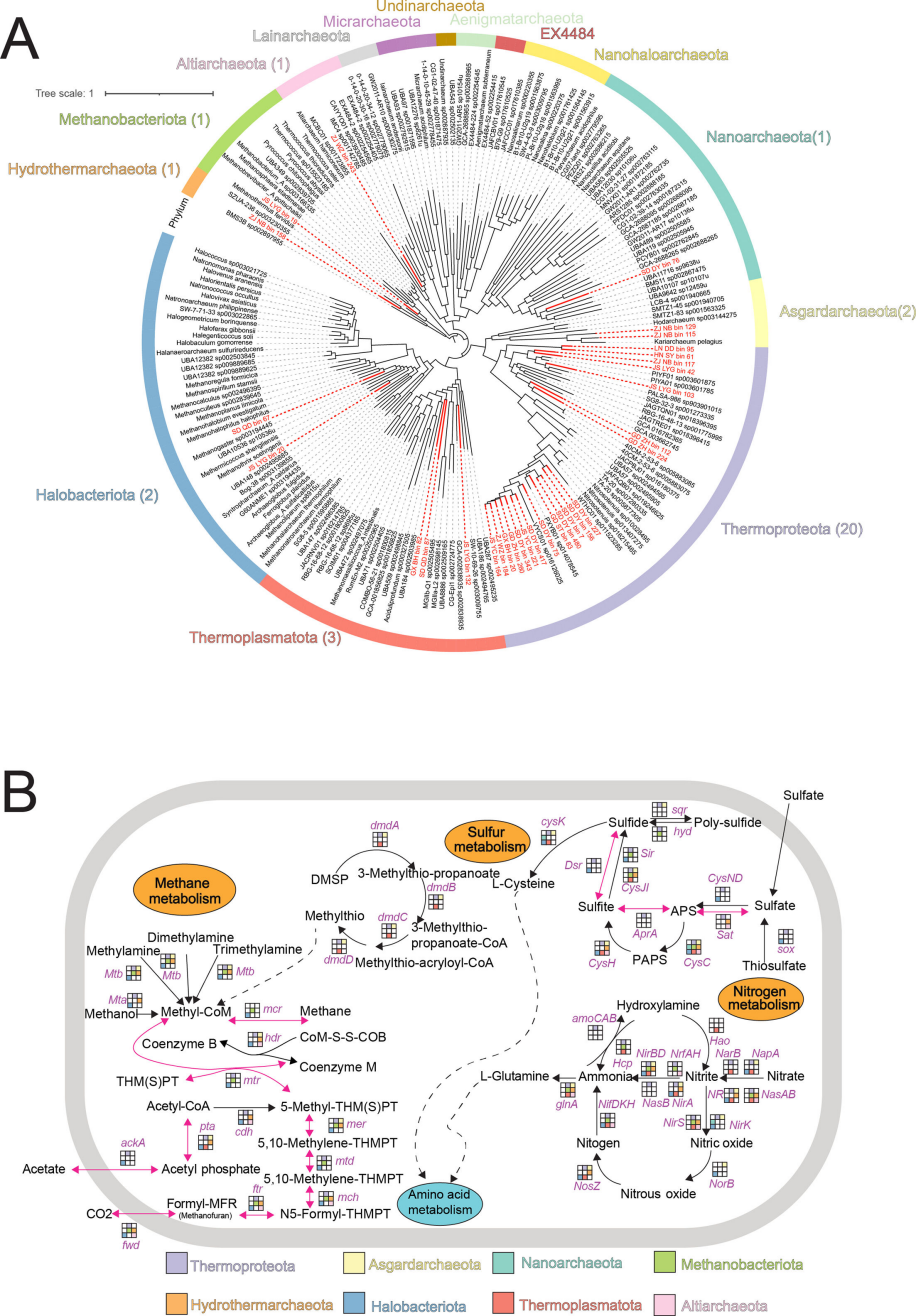
The phylogenetic relationships of recovered intertidal archaeal MAGs with known reference genomes and the functional potential carried by them. (**A**) Phylogenetic assignment of intertidal archaeal MAGs in the archaeal lineages. The phylogenetic tree was constructed based on 53 marker genes, with 195 recruited reference archaeal genomes. (**B**) Functional potential of intertidal archaeal MAGs in mediating nitrogen, sulfur, and methane cycles. Different colors represent the carrying of functional genes by different archaeal phyla. Blank indicates the absence of corresponding genes in the phyla.

With a big sampling effort, 144 sediment samples were collected from mudflat intertidal zones across China, ranging from the southernmost location of Sanya (SY) (18° 16′ 13″ N, 109° 40′ 51″ E) to the northernmost site of Dandong (DD) (39° 48′ 43″ N, 123° 41′ 37″ E) (Fig. 1; Data S1). All samples were subjected to archaeal 16S rRNA gene amplicon sequencing and 96 to shotgun metagenomic sequencing. In terms of taxonomy, *Thaumarchaeota*, *Euryarchaeota*, and *Crenarchaeota* were the dominant groups comprising the archaeal communities in the mudflat intertidal zone, as revealed by amplicon sequencing, shotgun metagenomic reads, and contigs (Fig. 2A). Notably, amplicon sequencing was remarkably different from shotgun metagenomes in profiling archaeal communities, especially in detecting *Euryarchaeota*, *Crenarchaeota*, and less abundant archaeal phyla (Fig. 2A). Shotgun metagenomes from the same sampling site were co-assembled to generate high-quality contigs via a multisample assembly effort. As a result, 31 high-quality archaeal MAGs were obtained from the 12 sampling sites. Through the Genome Taxonomy Database toolkit (GTDB-Tk, a standardized genome-based taxonomic framework), these 31 MAGs were categorized into 8 different archaeal phyla (Fig. 3A; Data S3), demonstrating a vast diversity of archaeal communities in the mudflat intertidal zones.

### Functional potential carried by intertidal archaeal MAGs

Multiple functional gene/orthologous databases, including NCycDB, MCycDB, SCycDB, and KEGG, were employed to probe the functional potential carried by intertidal archaeomes (Data S4 and S5). Although highly variable in taxonomic composition, the functional potential carried by the archaeal communities remained relatively stable (Fig. 2; Fig. S1). A variety of metabolic pathways were identified to be potentially mediated by these recovered archaeal MAGs. For instance, gene families involved in multiple N cycling processes, including assimilatory nitrate reduction, dissimilatory nitrate reduction, denitrification, and nitrification, were carried by the archaeal MAGs (Fig. 3B). Notably, the *Nitrososphaeria* MAGs can be further classified into 4 ammonia monooxygenase “super clades”: 8 NP-γ, 1 NP-α, 2 NP-ζ, and 2 NP-δ MAGs (Fig. S2C and Data S7). Of these, the NP-γ group exhibited a near-complete glycolysis pathway—a core module for three-carbon compound metabolism (Fig. S2D). For sulfur cycling, gene families involved in the linkages between inorganic and organic sulfur metabolism, organic sulfur transformation, and assimilatory sulfur reduction exhibited high relative abundance (Fig. 2B). Specifically, pathways of DSR, assimilatory sulfate reduction (ASR), and dimethylsulfoniopropionate (DMSP) metabolism were identified in multiple archaeal MAGs (Fig. 3B). For methane metabolism, gene families involved in aceticlastic methanogenesis, the central methanogenic pathway, and anaerobic oxidation of methane (AOM, defined as reverse methanogenesis coupled to alternative electron acceptors) exhibited high relative abundance (Fig. 2B). Four complete methanogenesis pathways, namely, methanogenesis via acetate, methanol, methylamine/dimethylamine/trimethylamine, and CO_2_, were identified in *Halobacteriota* and *Methanobacteriota* MAGs. In addition, genes related to AOM, including reverse electron transport components (e.g., Fpo-related genes), were identified in a Halobacteriota MAG. Based on these genomic features, JS_LYG_bin_19 and SD_QD_bin_67 were assigned as methanogen-type MAGs, whereas JS_LYG_bin_20 was assigned as an AOM-associated MAG (Fig. 3B; Fig. S4). Such results demonstrated the important roles that archaea play in mediating the biogeochemical pathways in intertidal mudflats.

### Horizontal gene transfer in intertidal archaeal genomes

**Fig 4:**
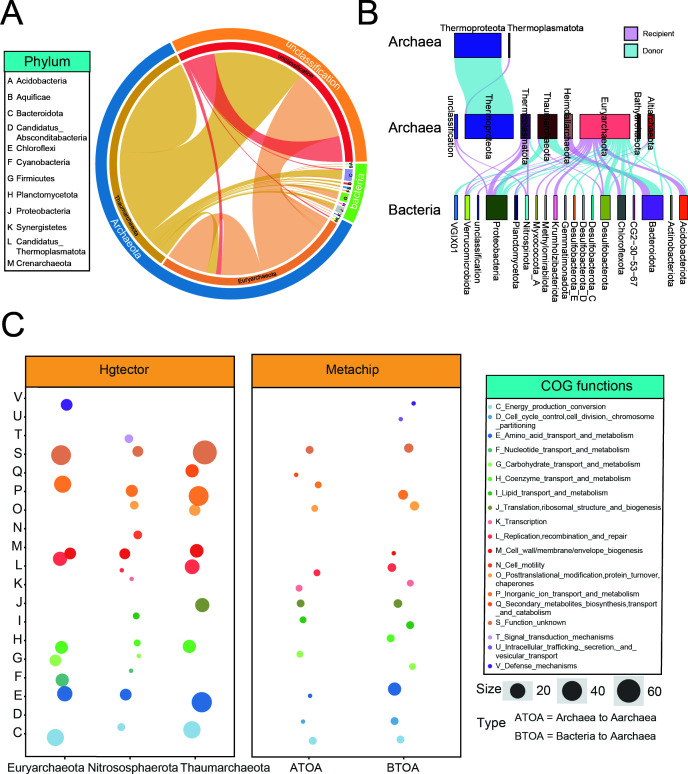
Horizontal gene transfer in intertidal archaeal MAGs. (**A**) Horizontal gene transfer events between intertidal archaeal MAGs and NCBI reference genomes. HGT events between different microbial phyla are displayed, with the width of the bands representing the number of HGT events. The program Hgtector was used to detect HGT events. (**B**) HGT events between the recovered intertidal archaeal MAGs and bacterial MAGs. The program Metachip was used to identify HGT events among MAGs. The width of the lines represents the HGT occurrence rate between different microbial phyla. Blue lines represented HGT from archaea to bacteria, while purple lines represented archaeal recipients. (**C**) Classification of horizontally transferred genes against the COG database. Different colors represent different COG categories, and different circle sizes represent the number of HGT events.

Besides their functional potential, we also explored the events of horizontal gene transfer in intertidal archaeal MAGs, aiming to resolve the genetic exchange and origin of functional genes therein and its effects on archaeal functions. Two different approaches were employed, including comparing against NCBI reference genomes and among the recovered intertidal prokaryotic MAGs (Data S8). First, the 31 archaeal MAGs were compared against 40,310 NCBI reference genomes using Hgtector (25), showing a total of 577 HGT events. In *Thaumarchaeota*, a total of 300 HGT events were found, of which 111 originated from archaea (including 95 from *Thaumarchaeota* itself), 32 from bacterial phyla, and 157 uncharacterized. In *Euryarchaeota*, 220 HGT events were identified, of which 73 originated from archaea (72 from *Euryarchaeota* itself), 22 from bacteria, and 125 uncharacterized (Fig. 4A). Functional assignment suggested that these HGT events primarily involved genes associated with inorganic ion transport and metabolism and amino acid transport and metabolism. Strikingly, in a *Methanosarcinia* MAG (JS_LYG_bin20), HGT events were observed for genes (K00030, K15231, and K01681) involved in the citrate cycle pathway. Second, HGT events among these 31 archaeal and the recovered intertidal bacterial MAGs were analyzed through the MetaCHIP approach (26). The results revealed a total of 62 HGT events between archaeal and bacterial MAGs, of which 27 were from bacteria to archaea (Fig. 4B). Similarly, HGT events were primarily detected in *Thaumarchaeota* and *Euryarchaeota* MAGs and mainly associated with amino acid transport and metabolism, inorganic ion transport and metabolism, posttranslational modification, protein turnover, and chaperones (Fig. 4C). Specifically, in a Thermoplasmata MAG (GX_BH_bin82), multiple HGT-derived genes assigned to amino acid metabolism, as well as the DNA repair gene *uvrA*, were detected. In both approaches, essential functional genes were obtained via HGT by archaeal MAGs. Such results suggested that archaeal HGT mainly occurred within the same domain and contributed remarkably to the archaeal functional potential.

### Spatial scaling of intertidal archaeal biodiversity

**Fig 5:**
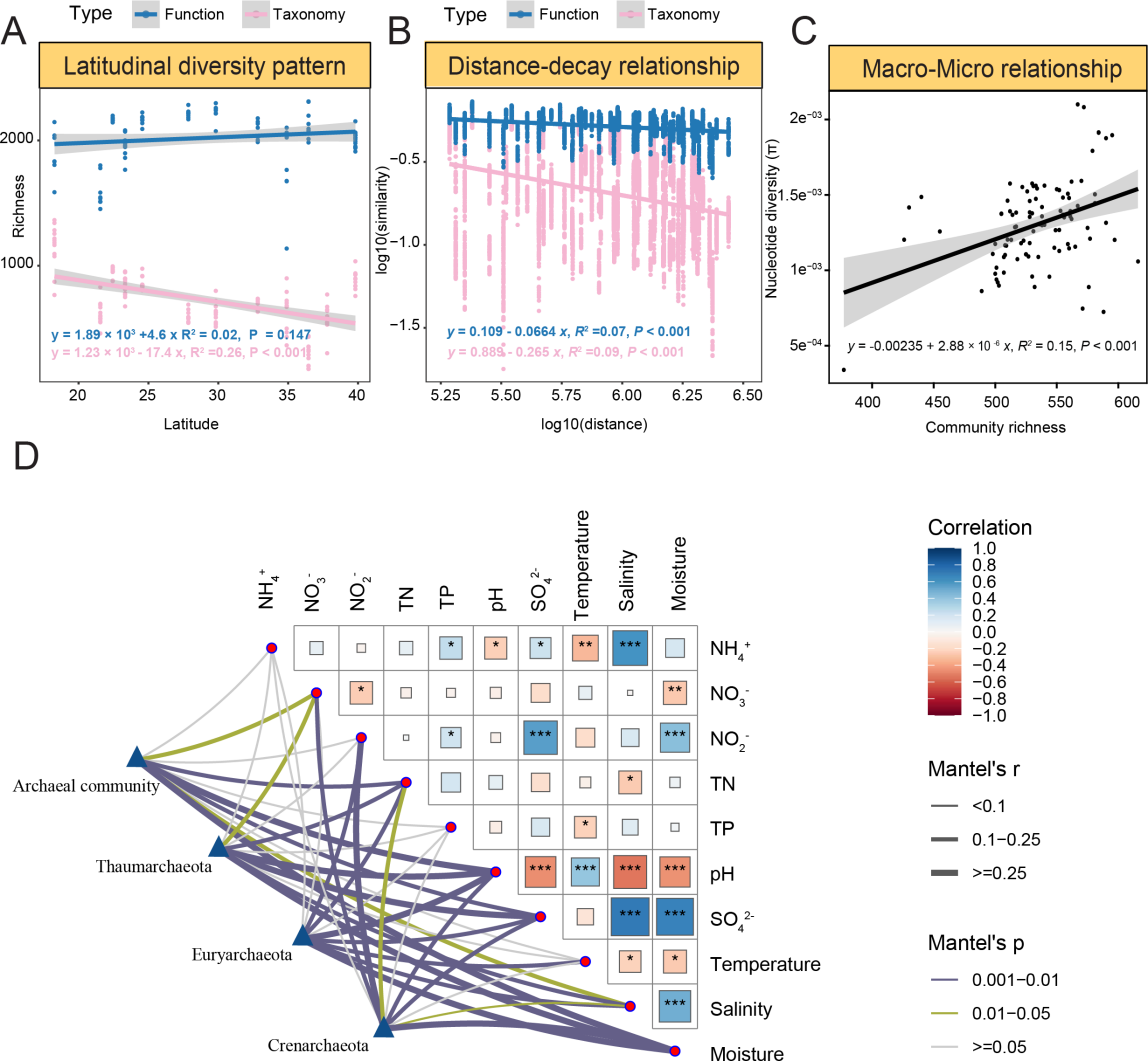
Spatial scaling of intertidal archaeal biodiversity and environmental drivers. (**A**) Latitudinal diversity patterns of archaeal taxonomy and function. (**B**) Distance-decay relationship (DDR) of archaeal taxonomy and function. As shown in panels A and B, the 16S amplicon sequencing was used for taxonomic profiling. Functional profiles were generated against the KEGG database. (**C**) The association between archaeal nucleotide diversity and community richness. (**D**) Partial Mantel analyses showing the associations between archaeal communities and environmental factors. In the figure, statistical significances are indicated by the following: *, *P* < 0.05; **, *P* < 0.01; ***, *P* < 0.001.

The large geographic sampling effort allowed us to explore the spatial scaling of intertidal archaeomes. To begin with, we analyzed the compositional and diversity variations of intertidal archaeomes along the latitudinal gradient. Clear latitudinal patterns in relative abundances were observed for major archaeal phyla such as *Thaumarchaeota* and *Euryarchaeota* (Fig. 2A). Specifically, the relative abundance of *Thaumarchaeota* peaked at mid-latitude (e.g., Ningbo), where the relative abundance of *Euryarchaeota* bottomed. In contrast, no patterned variation in archaeal functional genes was observed (Fig. S1). Latitudinal diversity gradient (LDG), which describes the pattern of increased species diversity toward the equator (27), was observed for the archaeal taxonomic diversity, though fluctuations were found for a few sites (Fig. 5A). Notably, significant LDG patterns were also observed for the three most abundant phyla, including *Thaumarchaeota*, *Euryarchaeota*, and *Crenarchaeota*, demonstrating LDG patterns across different archaeal lineages (Fig. S6A). In contrast, a stable distribution of archaeal functional traits along the latitude could be observed, resulting in insignificant latitudinal patterns (Fig. 5A; Fig. S1B).

Additionally, we also investigated the β-diversity patterns for intertidal archaeomes, which reflect the dissimilarity among different sites and are crucial for understanding the mechanisms mediating the differences in the composition of biological communities. First, the taxonomic compositions of intertidal archaeal communities clearly differed among different sampling sites, as shown by both amplicons and shotgun metagenomes (Fig. S5A and B). Consistent with the LDG patterns, the functional compositions were relatively more similar and indistinguishable among different sites (Fig. S5C). Second, the DDR was investigated, aiming to see how archaeal communities differed by geographic distance. Clear DDR patterns were observed for both taxonomic profiles (*d* = −0.265, *P* < 0.001) and functional traits (*d* = −0.0664, *P* < 0.001), of which the pattern for functional traits was much weaker (Fig. 5B). Of the three most abundant archaeal phyla, *Thaumarchaeota* showed the strongest DDR pattern, whereas much weaker DDR was observed for *Euryarchaeota* and *Crenarchaeota* (Fig. S6B).

Meanwhile, we also looked at the nucleotide diversity that measures the intra-population genetic variations by mapping reads to recovered archaeal contigs. High nucleotide-level variations could be observed for the recovered archaeal contigs and genes. For instance, a total of 104 variations were observed for the contig BH_K127_29654705, which encodes the Na+/H+ antiporter NhaC—an integral membrane protein. Among the variations, 81 occurred at the 3rd position on the codon, while 16 and 7 occurred at the 1st and 2nd positions, respectively. Such nucleotide variations were mainly observed at medium latitudinal sampling sites and were absent from sites in Sanya, Zhuhai, and Dongying (Fig. S7 and Data S9). No clear latitudinal pattern was observed for the archaeal nucleotide diversity. Two sampling sites, that is, Zhuhai and Xiamen, were found with much higher nucleotide diversity than the other sites (Fig. S8 and Data S10). Importantly, by correlating archaeal nucleotide diversity and community richness, a clear positive association was observed (*R*^2^ = 0.1, *P* = 0.002) (Fig. 5C). This suggested covariations between the community level macro-diversity and intra-population nucleotide-level micro-diversity.

### Ecological drivers of the intertidal archaeome biodiversity

**Fig 6:**
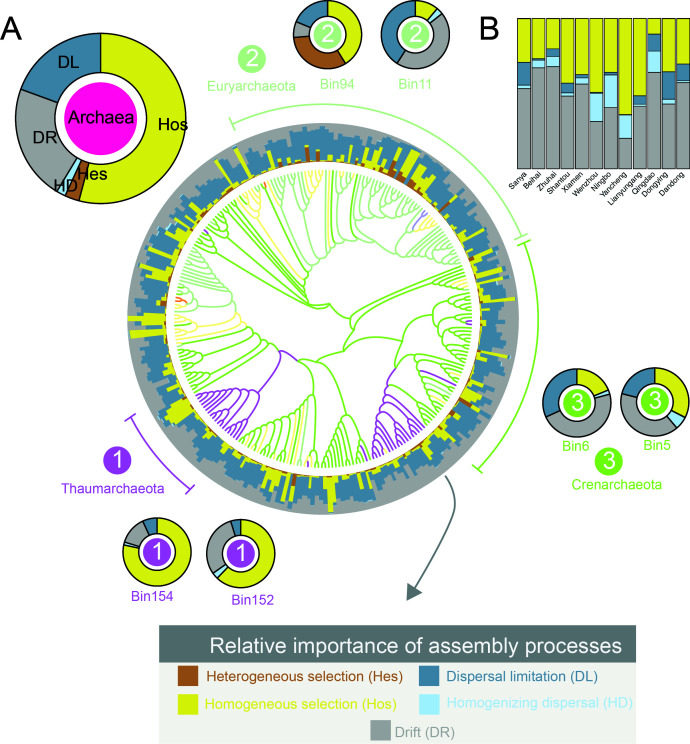
The importance of ecological processes in mediating intertidal archaeal communities. iCAMP was employed to quantify the relative importance of various ecological processes. (**A**) Both the overall community level and individual phylogenetic bins were analyzed. Individual phylogenetic bins, such as *Thaumarchaeota* (bin152 and bin154), *Euryarchaeota* (bin94 and bin11), and *Crenarchaeota* (bin5 and bin6), were extracted and are presented. (**B**) Assembly mechanisms of archaeal communities in the 12 sampling sites.

We then disentangled the ecological drivers that mediated the intertidal archaeal communities via a phylogenetic-bin-based approach (28). By analyzing βNTI and RC_bray_ metrics, the relative importances of ecological processes contributing to the assembly of archaeal communities were quantified. At the whole community level, homogeneous selection (HoS) (54.12%) was identified as the major process driving the variations in intertidal archaeal communities, followed by dispersal limitation (DL) (19.41%) and drift (DR) (21.98%) (Fig. 6A). Notably, different archaeal phylogenetic bins were mediated by dramatically different ecological processes (Data S11). For instance, the top two *Thaumarchaeota* bins (bin152 and bin154) were predominantly mediated by HoS, whereas the top two *Crenarchaeota* bins (bin5 and bin6) were mainly mediated by DR. In addition, the top two *Euryarchaeota* bins (bin94 and bin11) were mediated by completely different processes, that is, bin94 by HoS and HeS and bin11 by DR and DL (Fig. 6A). The assembly mechanisms of archaeal communities also exhibited a clear latitudinal variation. Across the national-scale intertidal samples, HoS and DR were the dominant processes. The role of HoS was particularly pronounced in the 28°N to 35°N latitudinal zone, indicating a shift in community assembly from stochastic to deterministic dominance. Among all sites, Yancheng demonstrated the highest relative contribution of HoS (Fig. 6B and Data S11).

Considering the high contribution of environmental selection, we further investigated the associations between environmental parameters (Data S12) and community variations. Significant correlation coefficients were found between certain environmental factors and archaeal community variations. Specifically, pH and moisture were demonstrated to have the strongest associations with archaeal community variations (*P* < 0.01), followed by NO_3_^−^, total nitrogen (TN), sulfate (SO_4_^2−^), and salinity (*P* < 0.05). For individual archaeal phyla, SO_4_^2−^, pH, and moisture were strongly associated with *Thaumarchaeota* (*P* < 0.01) and *Euryarchaeota* (*P* < 0.01), whereas NO_2_^−^ and moisture were strongly correlated with *Crenarchaeota* (*P* < 0.01) (Fig. 5D).

## DISCUSSION

Archaea are the essential biological components in the Earth’s biosphere. Over the past two decades, big progress has been made toward our understanding of the archaeal members in various environments (3), especially the clues showing the potential origin of eukaryotes (29) and the critical roles they play in mediating various biogeochemical cycling (30, 31), though the majority of them remain uncultivated. In the present research, the biological information of archaeal communities in mudflat intertidal zones was recovered via culture-independent approaches, specifically amplicon sequencing of 16S rRNA genes and shotgun metagenome sequencing of the total environmental DNA. Both the macro- and micro-diversity of intertidal archaeomes were investigated, showing how the archaeal communities were distributed and structured in the dynamic intertidal ecosystems from different angles.

In a previous study, clear latitudinal patterns for archaeal diversity and distributions have been observed in the estuarine ecosystem. Specifically, *Thaumarchaeota* predominates in low-latitude estuaries, *Bathyarchaeota* at mid-latitudes, and *Euryarchaeota* at high latitudes (32). Here, biogeographical patterns were also observed for the distribution of the major archaeal taxonomic groups along the Chinese intertidal zones, with marked differences deviating from estuary ecosystems. It was found that *Thaumarchaeota* peaked in medium-latitude intertidal zones such as Wenzhou, Ningbo, and Yancheng, whereas *Euryarchaeota* and/or *Crenarchaeota* predominated at lower and higher latitudes. Notably, remarkable differences were also observed between the relative abundance of archaeal taxa, especially for *Euryarchaeota*, *Crenarchaeota*, and *Thaumarchaeota* in a few sampling sites. The abundantly detected *Crenarchaeota* by amplicon sequencing was lowly profiled by shotgun metagenomes, whereas the many less abundant archaeal phyla detectable by shotgun metagenomes were rarely detected by amplicon sequencing. Such differences shall be due to the technical differences in recovering archaeal communities, especially the potential coverage and bias issues associated with 16S rRNA gene primers (33).

Similar to that previously observed for the whole microbial system (34), clear functional redundancy was also observed for the intertidal archaeal communities. The highly varied taxonomic composition at the phylum level along the coastal region resulted in the stable distribution of functional traits, either at pathway or functional gene levels, as well as for specific processes such as N, S, and methane cycling. Such discrepancy demonstrates decoupled archaeal taxonomy and function in natural ecosystems (35) and that the taxonomic groups are selected by environment because of the functions they carry (36, 37).

Although much effort has been made to recover archaeal genomes via metagenomic assembly, only 31 dereplicated high-quality archaeal MAGs were recovered, mainly in light of the high complexity of microbial communities in intertidal zones. However, the recovered MAGs spanned a wide range of archaeal phylogenetic diversity, covering archaeal taxa belonging to multiple lineages. Functional annotation of the genes carried by these MAGs demonstrated potentially critical roles they play in various biogeochemical cycles, such as C, N, S, and methane (3, 11). For instance, near-complete DSR and complete ASR pathways were detected in Thermoproteota, whereas Halobacteriota was found with a complete ASR pathway (9, 38). For methane production, *Euryarchaeota* play a key role in this process (7). Here, four methane production pathways were detected, with the acetate-dependent pathway being more abundant in terms of gene abundance. All four methane production pathways were detected in *Halobacteriota*, while only the CO₂ fixation pathway for methane production was found in *Methanobacteriota*, suggesting that *Halobacteriota* might consist of major archaeal taxa contributing to methane production in the mudflat intertidal ecosystem. However, as previously depicted, culture-dependent physiological and biochemical experimental evidences are needed to validate the functional activity carried by these archaeal taxa (39).

Compared to bacteria, our understanding of horizontal gene transfer in archaea is much lagging behind (40, 41). In this study, the HGT events were investigated for the recovered archaeal MAGs, both against NCBI reference genomes and against the recovered intertidal MAGs. In both cases, the majority of HGT events were detected among archaeal genomes, suggesting that HGT is more common among closely related organisms (42). Interestingly, for HGT between archaeal and bacterial MAGs, the ratios of archaea as donors and recipients were generally equal, suggesting that the genetic exchange between archaea and bacteria is bidirectional and likely nonpreferential. These results quite contrasted those of a former research revealing that the number of genes acquired by archaea from bacteria through HGT far exceeds the number of genes acquired by bacteria from archaea (43). As previously described, horizontally transferred genes associated with cell envelope biogenesis may help archaea better adapt to complex ecological environments (41). Here, we found that horizontally transferred genes significantly contributed to archaeal genomic functional diversity, particularly processes such as amino acid metabolism, inorganic ion transport, and energy conversion. In a *Methanosarcinia* MAG (JS_LYG_bin_20) belonging to *Euryarchaeota*, genes involved in the essential steps of citrate cycle were all acquired through HGT, demonstrating the importance of HGT to archaeal physiochemical activities. Therefore, the occurrence of HGT in archaea genomes not only reveals complex evolutionary dynamics but also provides crucial insights into their adaptability in various environments (41, 44).

Similar to many other studies (45–47), clear biogeographic patterns such as the well-recognized LDG and DDR were also observed for the intertidal archaeomes, for both taxonomic groups and functional genes. The spatial patterns observed for archaeal functional genes were, in general, weaker than those in taxonomic groups, as a result of functional redundancy properties in microbial systems (34). Meanwhile, the weak but still observable functional gene patterns also suggested potential functional partitioning of intertidal archaeomes along the latitudes. Notably, although a clear latitudinal pattern was not observed for the archaeal micro-diversity, the micro-diversity was significantly positively associated with macro-diversity, suggesting a close relationship between macro- and micro-diversity (48). Among various processes, homogeneous selection was mainly responsible for the diversity patterns of intertidal archaeomes, but their relative importance for individual groups substantially differed, demonstrating different adaptation strategies of archaeal groups in the intertidal ecosystem.

In conclusion, this study resolved both the macro- and micro-diversity of intertidal archaeal communities at a large geographic scale along the Chinese coasts. The intertidal archaeal phyla were highly varied, especially for the relative abundances of *Thaumarcheota* and *Euryarchaeota*, but were relatively stable considering the carried functional capacity, thereby maintaining essential ecosystem functions. The recovered archaeal groups collectively contributed to biogeochemical cycling in mudflat intertides, such that *Thaumarchaeota* are crucial for sulfur and nitrogen metabolism and *Euryarchaeota* for methane metabolism. HGT events, mainly identified among archaeal genomes, may have played critical roles in endowing ecosystem functions for different lineages, such as the citric acid cycle in *Methanosarcinia* and metabolism potential of various amino acids in *Thermoplasmata*. The diversity of intertidal archaeal communities was mainly structured by homogeneous selection, with different phylogenetic bins exhibiting distinct ecological processes. These findings collectively provided crucial insights into the biogeography, functional potential, and driving mechanisms of the dynamic intertidal archaeomes.

## MATERIALS AND METHODS

### Site description and sample collection

The intertidal zones are transitional areas between marine and terrestrial environments and hold extremely high biodiversity of both macro- and micro-organisms. In this study, mudflat intertidal sediment was selected to explore the community structure, functional potential, nucleotide morphology, and driving mechanisms of the archaeal communities therein. A total of 12 intertidal sites, geographically ranging from Sanya (southernmost) to Dandong (northernmost), were selected for sediment sample collection along the Chinese coastline (Fig. 1A). To adequately represent each site, 15 sediment samples were collected across different locations within the area. To reduce the influence of temperature on samples, the collection of samples was carried out from April (Sanya) to June (Dandong) 2021, balancing the temperature differences in south and north China. Exposed intertidal sediments at low tides were sampled at each site with 5 replicates (~15 cm depth), which were then thoroughly mixed. The homogenized sediment samples (approximately 200 g) were refrigerated and then transported to the laboratory.

### Physicochemical parameters and climatic factors

For the collected samples, a total of 10 physicochemical indicators were measured, including temperature, pH, salinity, TN, total phosphorus (TP), ammonia nitrogen (NH_4_^+^-N), nitrate nitrogen (NO_3_^−^-N), nitrite nitrogen (NO_2_^−^-N), SO_4_^2−^, and moisture content. These environmental parameters were measured using the method described in the article (49).

### DNA extraction and sequencing

Total DNA was extracted from 0.2 g of freeze-dried samples using the FastDNA SPIN Kit for Soil (MP Biomedicals, USA). DNA purity was assessed by measuring the absorbance ratios at 260/280 nm and 260/230 nm using a NanoDrop spectrophotometer (Nano Drop, Wilmington, DE). For each sampling site, 12 quality-approved samples were selected for archaeal 16S rRNA gene amplicon sequencing (primers: 524F-10-ext/Arch958R-mod), while shotgun metagenomic sequencing was performed for 8 samples (Illumina NovaSeq platform, paired-end 150 bp, with an average output of 20 Gb per sample).

### Sequencing data processing

For amplicon sequencing data, primers were removed using Cutadapt (v 4.1) (50) with the parameters -g TGYCAGCCGCCGCGGTAA -G CCGGCGTTGAVTCCAATT. The clipped 16S rRNA data were then processed using the DADA2 pipeline (51). Reads with an expected error rate higher than 1.5 (maxEE = [1.5, 1.5]) were discarded. The taxonomic annotation of amplicon sequencing variants (ASVs) was performed via DADA2’s assignTaxonomy module against the RDP database (August 2023 version). Finally, ASVs classified as archaea were filtered to generate archaea-specific ASV profiles.

For shotgun metagenome analyses, raw reads were quality-controlled using Trimmomatic (v 0.39) (52) with parameters (TRAILING 20, LEADING 20, and MINLEN 36). After data cleaning, MEGAHIT (v 1.2) (53) was used to assemble the data for 8 samples, and the resulting contigs were processed using 3 binning methods, MaxBin2 (v 2.2.7) (54), MetaBAT2 (v 2.2.15) (55), and CONCOCT (v 1.1) (56). Subsequently, the binning results were integrated using metaWRAP (v 1.3) (57), ultimately generating draft genomes. Taxonomic assignment for archaeal sequences was performed at three different levels, namely, reads, contigs, and MAGs. Specifically, Kraken2 was used to annotate raw reads and assembled contigs by employing the NT database. MMseq2 (v 16.0) (58) was employed to annotate assembled contigs by employing the GTDB database. MAG completeness was evaluated with CheckM (v 1.2) (59). MAGs (completeness >50% and contamination <10%) were preserved for taxonomic classification using GTDB-Tk (v 1.5.1) (60).

### Reconstruction of phylogenetic trees

To identify the taxonomic information of recovered archaeal MAGs, a total of 164 representative archaeal genomes were downloaded from NCBI, covering various phyla. By combining the reference genomes with the recovered archaeal MAGs, an archaeal genome data set was constructed. Using the classify_wf function of GTDB-Tk, marker genes were filtered, concatenated, and subjected to multiple sequence alignment with MAFFT (v 7.0) (61). The phylogenomic tree was inferred using the maximum-likelihood method in IQ-TREE (v 2.1.2) (62) (model: LG + F + R10), with 1,000 bootstraps and 1,000 approximate likelihood-ratio tests. For the classification of *Bathyarchaeia* archaea and the sub-classification of *Nitrososphaeria* archaea, we collected 125 and 49 representative genomes, respectively. Phylogenomic trees were constructed under the LG +F + R8 and insect + F + R6 models, with both trees visualized using iTOL (63).

### Functional annotation of archaeal genes

Archaeal genes were first predicted from the assembled archaeal contigs. To minimize homologous interference, redundant protein sequences were grouped and removed through CD-HIT (v 4.6.8 ) (64) with parameters -c 0.6, -as 0.8, and -n 3. Subsequently, functional annotation of the nonredundant protein set was conducted using a series of functional gene databases including NCycDB (65), SCycDB (66), McycDB (67), and KEGG (68) databases. Homologous searching against NCycDB, SCycDB, and MCycDB was carried out using DIAMOND (69) (v 2.1.10) (parameters: -k 1 -e 0.0001 -p 30) to obtain the best mappings, while KEGG annotation was carried out using GhostKOALA (70) to assign KEGG Orthology (KO) identifiers to predicted genes, as well as further analysis of metabolic pathways. Gene abundance was calculated using the count mode in CoverM (71) (with outliers removed based on the mean values reported by CoverM). To eliminate the influence of varying sequencing depths on downstream analyses, gene abundance profiles were rarefied using the rarefy function in the vegan package in R. The depth of all samples was rarefied to the minimum (i.e., the smallest total read count across samples) by randomly subsampling a fixed number of sequences. This approach simulates gene composition at a uniform sequencing depth, ensuring comparability among samples in diversity analyses. Additionally, ammonia-oxidizing archaea were classified based on the amoA 2380 database (72). For MAGs, KO annotation was conducted with EggNOG-mapper (73) (v 2.1.12) to systematically characterize the central carbon metabolism of *Bathyarchaeia* and *Nitrososphaeria* archaea. The metabolic pathways of MAGs were analyzed and visualized using heatmaps, with tools such as KEGG and KofamKOALA (74), along with customized Python scripts.

### Identification of horizontal gene transfer events

To identify potential HGT in the recovered intertidal archaeal MAGs, two approaches were employed: HGTector (v 2.0) and MetaCHIP (v 1.10). HGTector utilizes a pre-built database (2023 version) comprising 129,809,746 distinct protein sequences across 40,310 microbial genomes and employs the DIAMOND program (parameters: --evalue 1e^−20^ --query-cover 50% --approx-id 30% --max-target-seqs 500). To explore HGT events between intertidal archaea and bacteria, as well as among archaea themselves, we constructed a separate database for intertidal archaeal and bacterial MAGs. The analysis was conducted using MetaCHIP. Functional assignment for the detected HGT genes was done by eggNOG-mapper for COG classification, and the results were visualized using the ggalluvial (75) (v 0.12.5) and ggplot2 (76) (v 3.5.1) packages in R (v 4.02).

### Spatial scaling pattern and micro-diversity analyses

The LDG and DDR were investigated to assess the distribution of archaeal functional genes and taxonomic groups in this study. LDG was analyzed using linear regression between latitude and species richness, showing the relationship between archaeal diversity and latitude. DDR was analyzed through linear regression between geographic distance and community similarity. In addition to HGT, we also calculated nucleotide diversity (π values) using the Metapop (77) (v1.0) tool and analyzed the relationship between macro-diversity (richness) and micro-diversity (π values) using linear regression.

### Environmental factors and community assembly processes

To investigate the roles of environmental factors in shaping the compositional variations of taxonomic groups and functional genes of intertidal archaeomes, the partial Mantel test was employed using the linkET (v 0.0.7.4). To infer the community assembly mechanisms, iCAMP (v 1.5.12) was employed to analyze the assembly processes of archaeal community compositions. The relative importance of different ecological processes was quantified, including deterministic processes (e.g., heterogeneous and homogeneous selection) and stochastic processes (e.g., dispersal limitation, homogenizing dispersal, and ecological drift). To analyze the assembly mechanisms of the archaeal community at different scales (the overall archaeal community, different locations, and individual groups), the phylogenetic signal threshold was set to 0.2, with a minimum bin size of 48. By screening the contributions of each bin, the assembly processes for dominant bins in each phylum were analyzed. Phylogenetic trees were constructed and visualized using iTOL.

## Data Availability

The metagenomic data used in this research are available in the NCBI SRA database, under project ID PRJNA957716. Data S1 contains detailed descriptions of the metagenomic and amplicon data sets. Representative ASVs, contigs, MAGs, and environmental factors can be accessed via https://doi.org/10.5281/zenodo.15322441. The statistical analysis and visualization scripts are available on GitHub at https://github.com/hahamark-learn/Macro-and-micro-biodiversity-of-mudflat-intertidal-archaeomes-along-the-Chinese-coasts.

## SUPPLEMENTAL MATERIAL

Data S1 msystems.01434-25-s0001.xlsx Geographic information and sequencing statistics for the collected mudflat intertidal samples.

Data S2_1 msystems.01434-25-s0002.xlsx Archaeal taxonomic profiles based on ASVs.

Data S2_2 msystems.01434-25-s0003.xlsx Archaeal taxonomic profiles based on ASVs.

Supplemental figures msystems.01434-25-s0004.docx Figures S1 to S8.

Data S3 to S6 msystems.01434-25-s0005.xlsx Additional information.

Data S7 to S12 msystems.01434-25-s0006.xlsx Additional information.

ASM does not own the copyrights to Supplemental Material that may be linked to, or accessed through, an article. The authors have granted ASM a non-exclusive, world-wide license to publish the Supplemental Material files. Please contact the corresponding author directly for reuse.
