# Supplementary material for: Patterns and drivers of macro- and micro-diversity of mudflat intertidal archaeomes along the Chinese coasts
Source: mSystems. 2026 Mar 27;11(4):e01434-25. doi: 10.1128/msystems.01434-25 (PMC13098193; doi:10.1128/msystems.01434-25)
Supplement: Supplemental figures — Figures S1 to S8. [file msystems.01434-25-s0004.docx]

**Supporting Information for**

**Patterns and drivers of macro- and micro-diversity of mudflat intertidal archaeomes along the Chinese coasts**

Yan Li^1^, Mengzhi Ji^1^, Qichao Tu^1,2,^*

^1^ Institute of Marine Science and Technology, Shandong University, Qingdao, China

^2^ Qingdao Key Laboratory of Ocean Carbon Sequestration and Negative Emission Technology, Shandong University, Qingdao, China

^*^ Correspondence should be addressed to Qichao Tu, Email: tuqichao@sdu.edu.cn


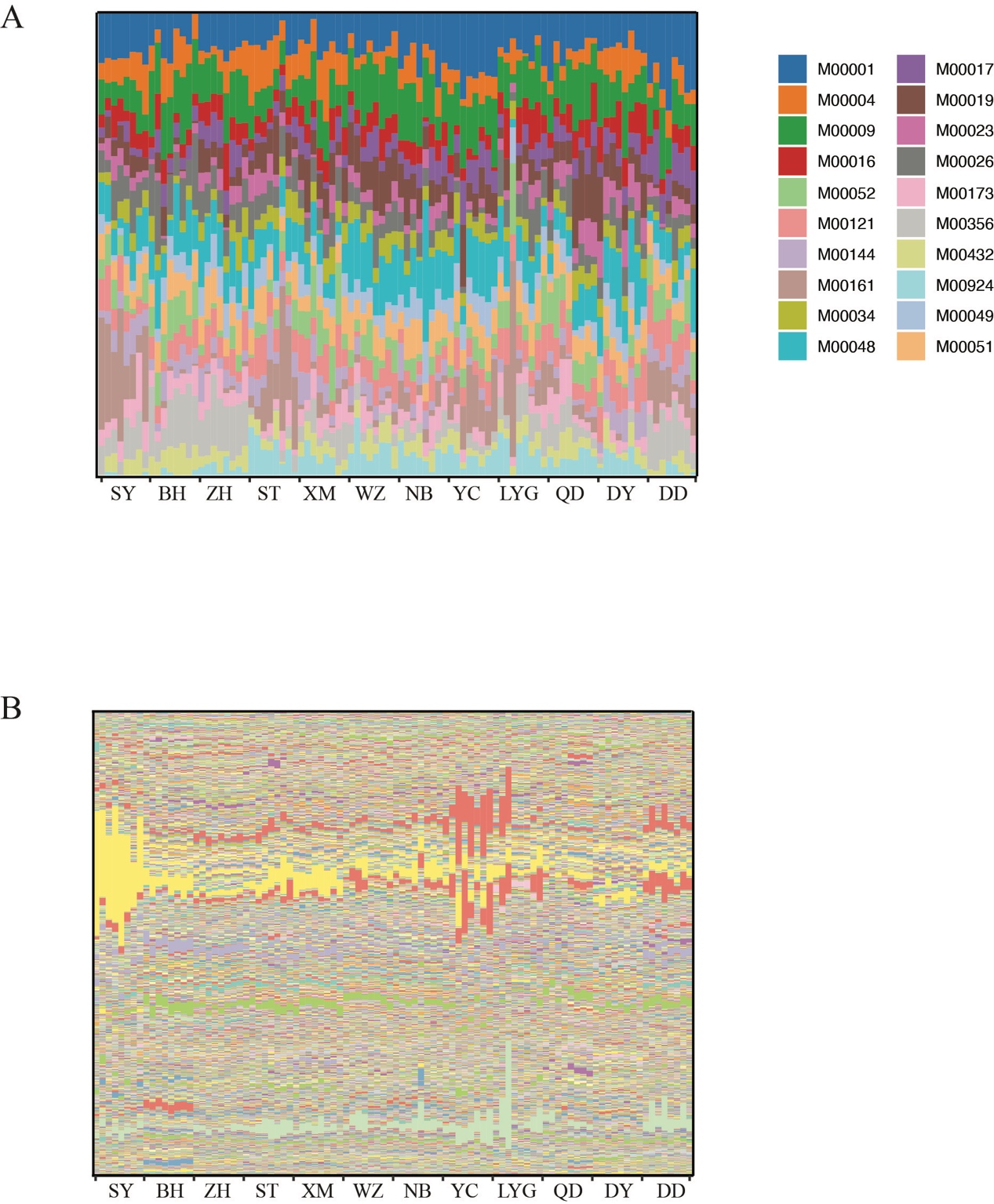


**Supplementary Figure 1. Functional composition of intertidal archaeal communities based on KEGG pathway and ortholog annotations.** **(A)** The functional composition of intertidal archaeomes at the pathway level. **(B)** The functional composition of intertidal archaeomes at the KEGG ortholog (KO) level. In panels A and B, functional profiles were generated by annotating the recovered archaeal contigs against the KEGG database. For KEGG pathways in panel A: M00001: Glycolysis (Embden-Meyerhof pathway); M00004: Pentose phosphate pathway (Pentose phosphate cycle); M00009: Citrate cycle (TCA cycle, Krebs cycle); M00016: Lysine biosynthesis; M00017: Methionine biosynthesis; M00019: Valine/isoleucine biosynthesis; M00026: Histidine biosynthesis; M00048: De novo purine biosynthesis; M00051: De novo pyrimidine biosynthesis; M00053: Deoxyribonucleotide biosynthesis; M00088: Ketone body biosynthesis; M00121: Heme biosynthesis; M00144: NADH quinone oxidoreductase; M00159: V/A-type ATPase; M00173: Reductive citrate cycle; M00309: Non-phosphorylative Entner-Doudoroff pathway; M00356: Methanogenesis (conversion of methanol to methane); M00357: Methanogenesis (conversion of acetate to methane) M00432: Leucine biosynthesis; M00892: UDP-N-acetyl-D-glucosamine biosynthesis. KOs in panel C were not explicitly coded in the figure, as the main purpose was to show stable distribution of KOs across different sampling sites.

_
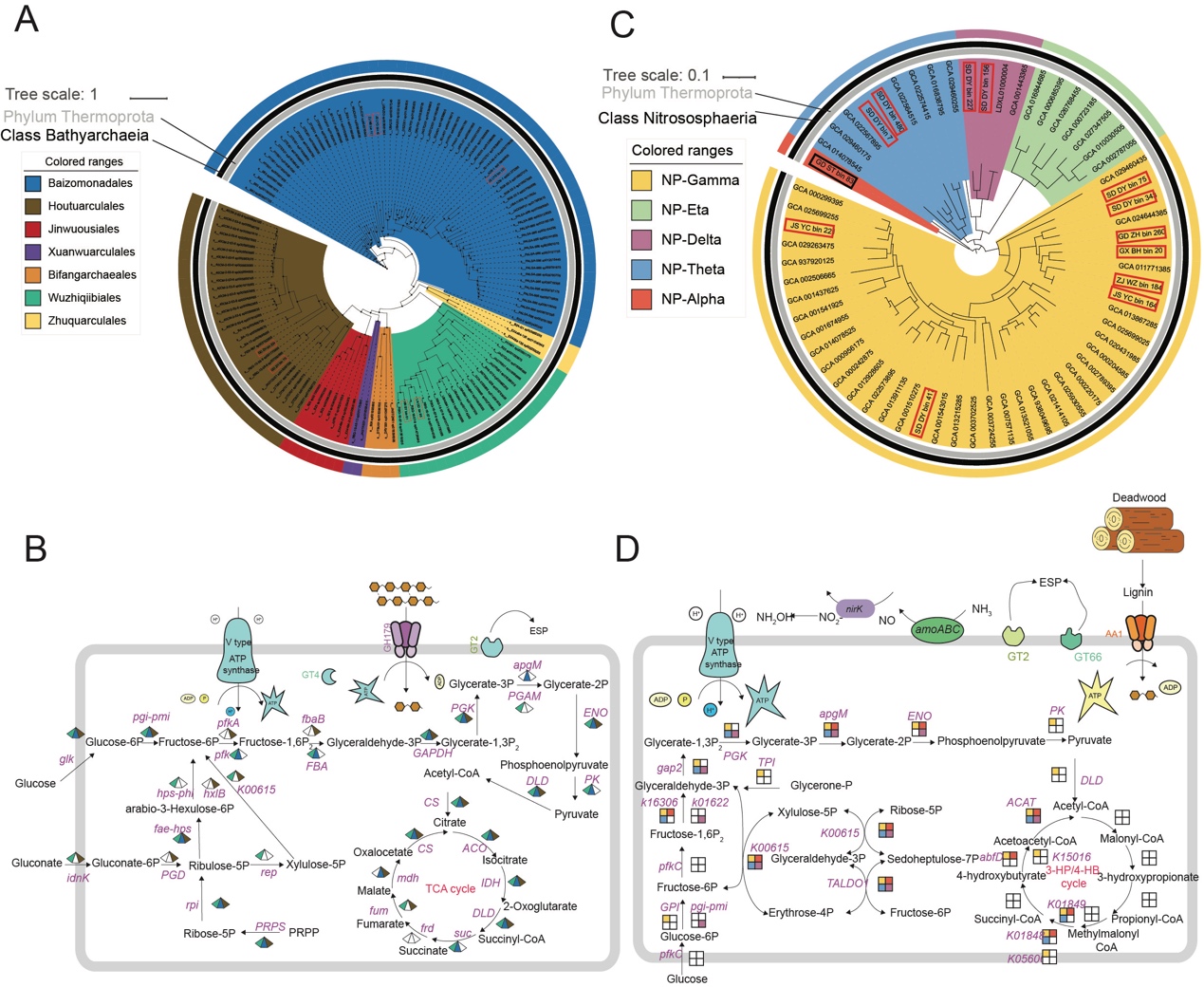
_

**Supplementary Figure 2. Phylogenetic position of the recovered *Bathyarchaeia* and *Nitrososphaeria* MAGs, and their carrying central carbon metabolic pathways. (A)** Phylogenetic relationship of the recovered *Bathyarchaeia* MAGs with reference genomes. The phylogenetic tree was constructed using 53 archaeal marker genes, by recruiting 132 reference genomes of *Bathyarchaeia*. **(B)** The central carbon metabolic pathways carried by *Bathyarchaeia* MAGs. **(C)** Phylogenetic relationship of the recovered *Nitrososphaeria* MAGs with reference genomes. The phylogenetic tree was constructed using 53 archaeal marker genes, by recruiting 62 reference genomes of *Nitrososphaeria*. **(D)** The central carbon metabolic pathways carried by *Nitrososphaeria* MAGs.


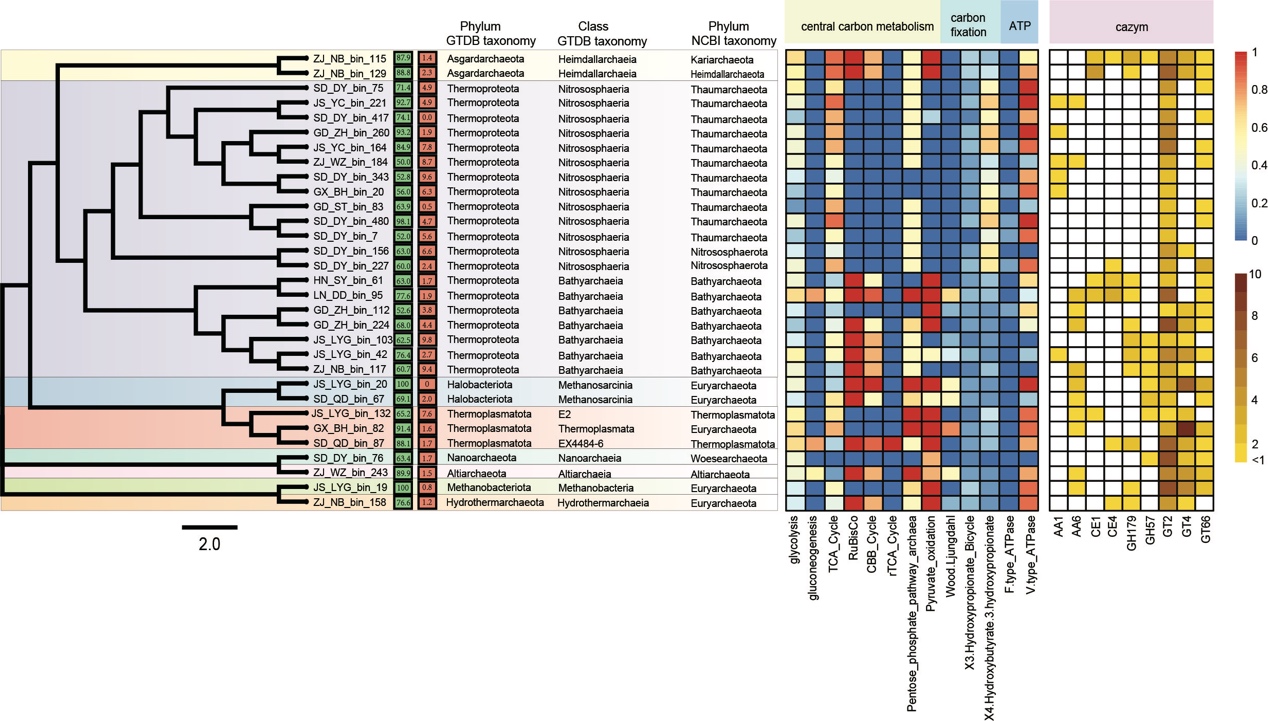


**Supplementary Figure 3. Carbon cycling pathways in archaeal MAGs.** In the left panel, a phylogenomic tree was constructed for the 31 recovered archaeal MAGs using the arc53 maker set. Information such as genome completeness (green) and redundancy (orange) was shown next to the MAG IDs. Both GTDB and NCBI taxonomic information was added. A total of 1000 bootstrap was used for tree construction. KeggDecoder heatmap was provided based on KEGG annotation. Carbon metabolic pathways inferred from KEGG and CaZyme database, including central carbon metabolism, carbon fixation, ATP, and CaZyme families, were displayed. A scale of 0 to 1 was used to describe the completeness of the metabolic process in each archaeal MAG. A scale of 0 to 10 was used to describe the number of CaZyme genes in each MAG.


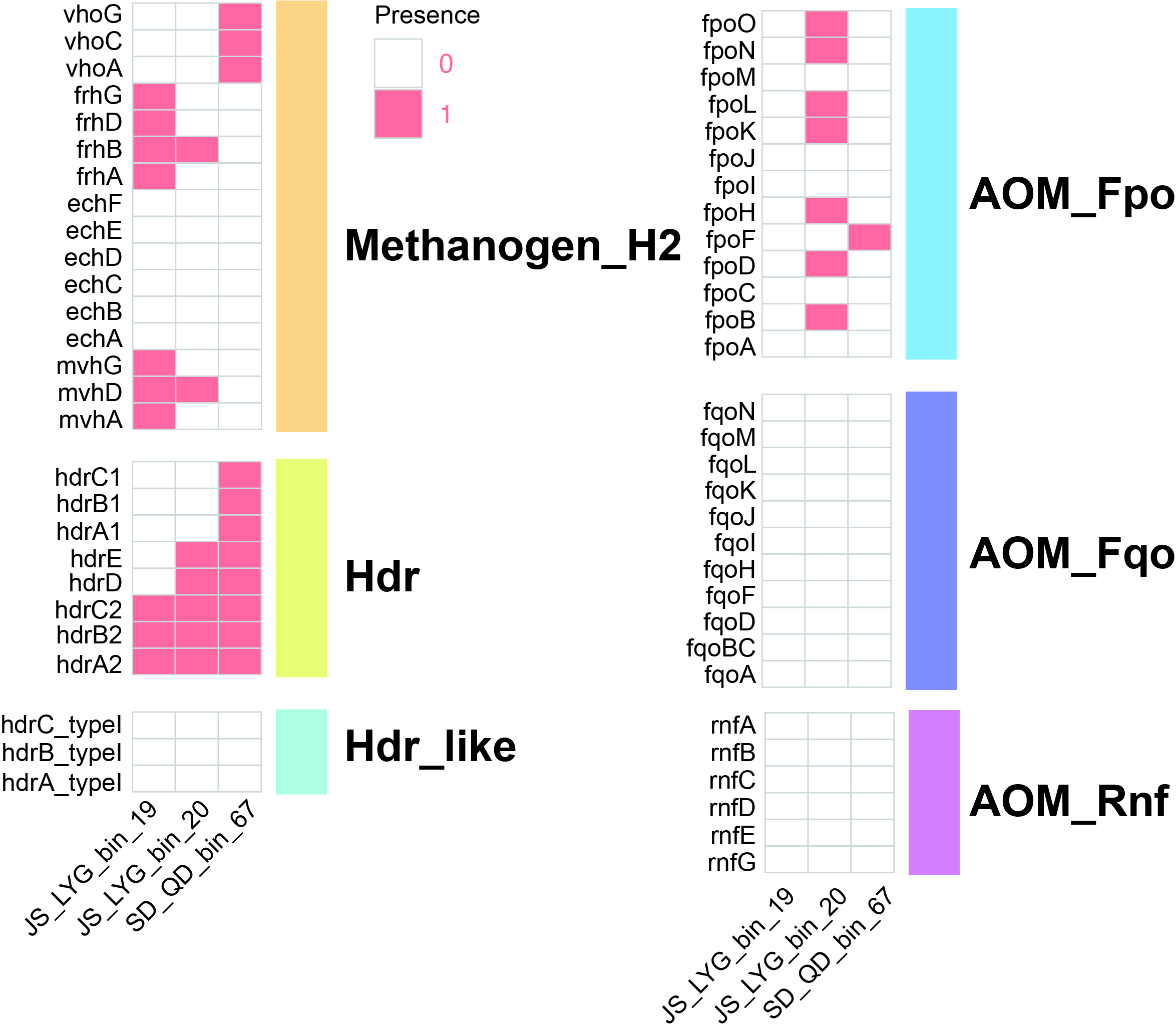


**Supplementary Figure 4. Gene presence–absence patterns of methanogenesis- and AOM-related pathways in three archaeal MAGs.** Heatmap showing the presence or absence of genes associated with methanogenic hydrogenase systems (Methanogen_H2), heterodisulfide reductase complexes (Hdr and Hdr-like), and reverse electron transport components related to anaerobic oxidation of methane (AOM_Fpo, AOM_Fqo, and AOM_Rnf). MAG names are shown on the x-axis.


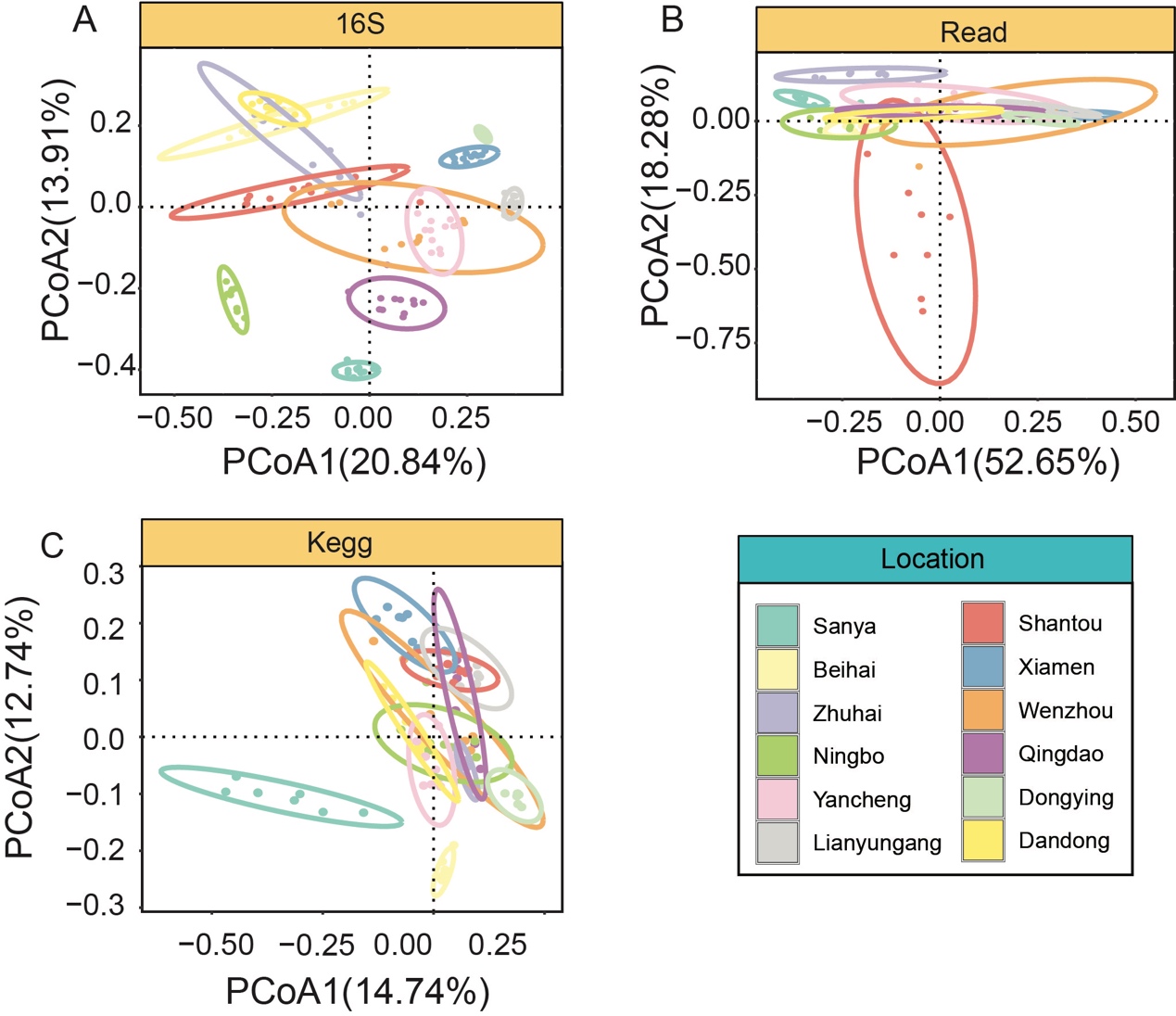


**Supplementary Figure 5. Ordination analyses of intertidal archaeal taxonomic (A and B) and functional gene compositions (C).**


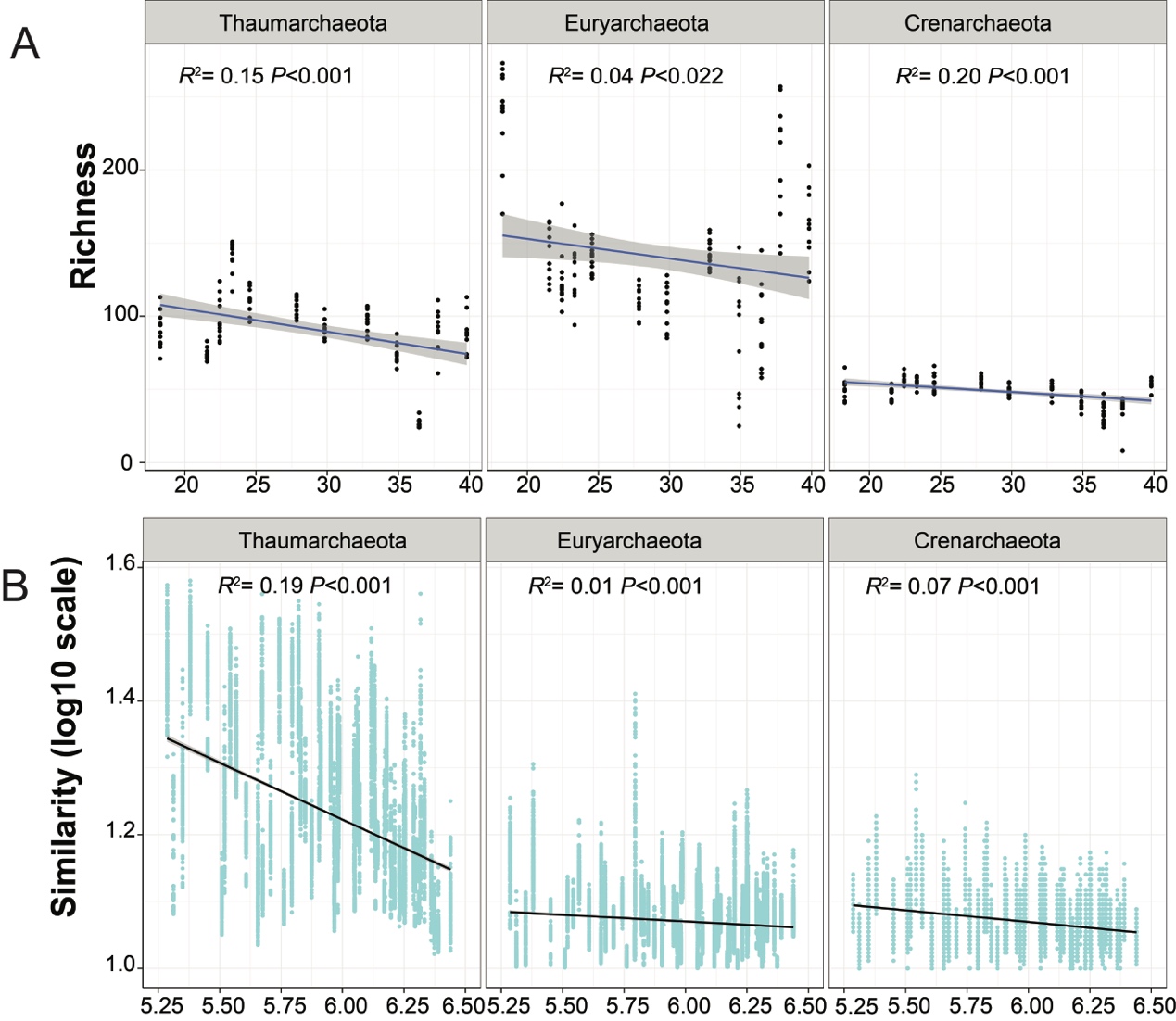


**Supplementary Figure 6. Spatial scaling of abundant archaeal phyla. (A)** Latitudinal diversity patterns of Taumarchaeota, Euryarchaeota and Crenarchaeota. **(B)** Distance-decay relationship of Taumarchaeota, Euryarchaeota and Crenarchaeota. Archaeal community profiles based on 16S rRNA gene amplicon sequencing were used for the analyses.


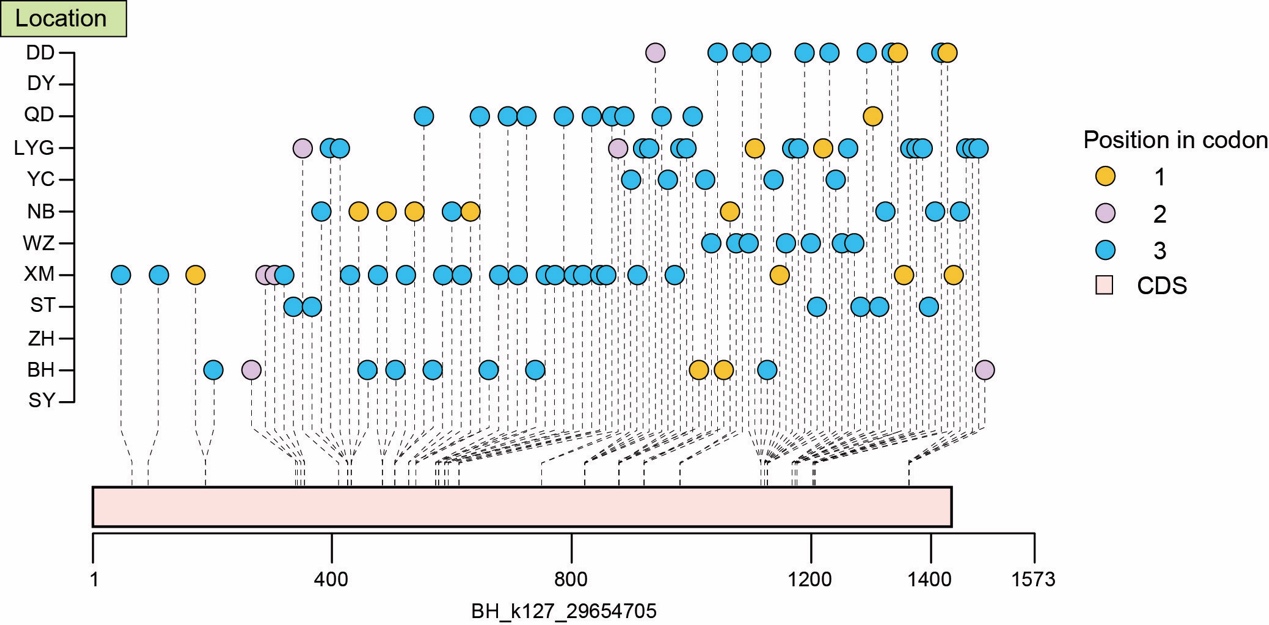


**­****Supplementary** **Figure 7. Nucleotide-level variation of the recovered contig BH_K127_29654705 across sampling sites.** The occurring positions on codon of the variations were illustrated by different colors. The contig carried a functional gene encoding the Na+/H+ antiporter NhaC—an integral membrane protein.

**
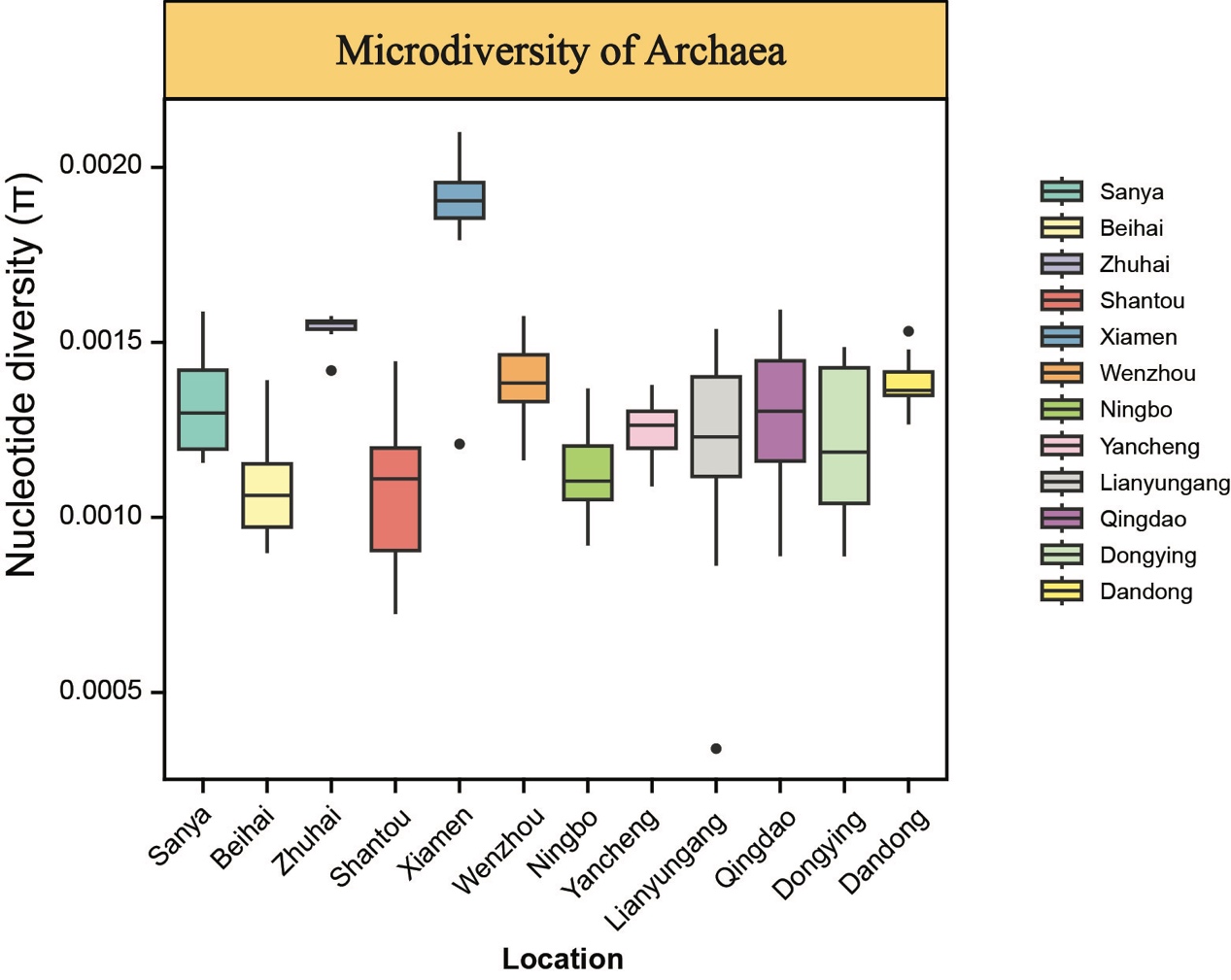
**

**Supplementary Figure 8. Spatial scaling of intertidal archaeal microdiversity.** Nucleotide diversity (π) of archaeal communities across the sampling intertides.
